# Sixteen years of ICPC use in Norwegian primary care: looking through the facts

**DOI:** 10.1186/1472-6947-10-11

**Published:** 2010-02-24

**Authors:** Taxiarchis Botsis, Carl-Fredrik Bassøe, Gunnar Hartvigsen

**Affiliations:** 1 Department of Computer Science, University of Tromsø, 9037 Tromsø, Norway; 2 Norwegian Centre for Electronic Medical Records, Institute of Neuromedicine, Faculty of Medicine, Norwegian University of Science and Technology, 7489 Trondheim, Norway; 3 Norwegian Centre for Integrated Care and Telemedicine, University Hospital of North-Norway, 9038 Tromsø, Norway; 4 Current address: Lyngveien 14b, 5101 Eidsvaagneset, Bergen, Norway

## Abstract

**Background:**

The International Classification for Primary Care (ICPC) standard aims to facilitate simultaneous and longitudinal comparisons of clinical primary care practice within and across country borders; it is also used for administrative purposes. This study evaluates the use of the original ICPC-1 and the more complete ICPC-2 Norwegian versions in electronic patient records.

**Methods:**

We performed a retrospective study of approximately 1.5 million ICPC codes and diagnoses that were collected over a 16-year period at 12 primary care sites in Norway. In the first phase of this period (transition phase, 1992-1999) physicians were allowed to not use an ICPC code in their practice while in the second phase (regular phase, 2000-2008) the use of an ICPC code was mandatory. The ICPC codes and diagnoses defined a problem event for each patient in the PROblem-oriented electronic MEDical record (PROMED). The main outcome measure of our analysis was the percentage of problem events in PROMEDs with inappropriate (or missing) ICPC codes and of diagnoses that did not map the latest ICPC-2 classification. Specific problem areas (pneumonia, anaemia, tonsillitis and diabetes) were examined in the same context.

**Results:**

Codes were missing in 6.2% of the problem events; incorrect codes were observed in 4.0% of the problem events and text mismatch between the diagnoses and the expected ICPC-2 diagnoses text in 53.8% of the problem events. Missing codes were observed only during the transition phase while incorrect and inappropriate codes were used all over the 16-year period. The physicians created diagnoses that did not exist in ICPC. These 'new' diagnoses were used with varying frequency; many of them were used only once. Inappropriate ICPC-2 codes were also observed in the selected problem areas and for both phases.

**Conclusions:**

Our results strongly suggest that physicians did not adhere to the ICPC standard due to its incompleteness, i.e. lack of many clinically important diagnoses. This indicates that ICPC is inappropriate for the classification of problem events and the clinical practice in primary care.

## Background

Medical standards are essential resources for clinical decision making and decision support, audit, governance, research, education and training [1]. Medical classifications are medical standards that are developed to facilitate the primary and secondary use of clinical data. The various versions of Systematized Nomenclature of Medicine (SNOMED), International Classification of Diseases (ICD) and International Classification for Primary Care (ICPC) are some examples of medical classifications.

ICPC was first published in 1987 by the WONCA (World Organization of Family Physicians) International Classification Committee (WICC) as a tool to order the domain of family practice in the format of episodes of care [2]. The current version (ICPC-2) is the outcome of many revisions over the first ICPC-1 version [3]. It has been translated in many languages and it is used as part of the primary care practice in several countries.

General practitioners (GPs) are often under-motivated to code their consultation data [4]. The quality of electronic patient record (EPR) data in primary care appears to be a major issue for computerized systems that utilize other terminologies as well, such as the Read clinical classification [5]. Porcheret *et al *studied the use of Read codes in a UK region and found that the coding completeness for all primary care centre consultations with a physician ranged from 5% to 97% between practices when the system did not demand a code for the storage of clinical narratives [6].

ICPC coding has been compulsory for all GPs in Norway since 1992 [7]. The Norwegian Centre for Informatics in Health and Social Care (KITH) maintains all the electronic versions of ICPC on behalf of WICC and supports the download of both the English and Norwegian versions [8]. In order to better cover the clinical needs, KITH extended the Norwegian ICPC-2 to include more diagnoses than the English version; thus, each code may correspond to more than one diagnosis for the same problem area.

The present study evaluates the ICPC use in primary care EPRs and focuses on missing and non-existing codes, and diagnoses that do not map the diseases, the symptoms or the procedures of the standard ICPC-2 classification. The extent of the problems with ICPC use was assessed by using a large data set that was collected over a 16-year period in Norway. Based on our findings various aspects are discussed and potential directions for future work are suggested.

## Methods

### PROMED

The data set that was used in this study was extracted from primary care **PRO**blem-oriented electronic **MED**ical records (PROMEDs); PROMEDs operated from 1984 to 2008. The first PROMED version (1984) did not include any disease classification and the diagnoses were entered manually by the physicians. This version was developed in Clipper 87 and Clipper 5 programming languages while the data was stored in dBASE databases.

The functionality for recording ICPC-1 diagnoses and codes was added to RPOMED system in 1992. The period from 1992 to 1999 was considered as a transition phase and physicians were allowed to reuse patient diagnoses (ICPC-adjusted or not) that were stored in the system before 1992 either associating them with an ICPC-1 code or not.

The PROMED version that was used in the regular phase (2000-2008) was built in Ms Visual Basic 6. The dBASE databases were automatically converted to a similar format in Ms Access 97 databases retaining the data that was collected in the period 1984-1999. The Access 97 databases were accessed using ActiveX Data Objects (ADO) and Structured Query Language (SQL). Both PROMED versions ran on personal computers that were interconnected over a Local Area Network (LAN).

PROMED included various modules for narratives, laboratory routines, drugs and prescriptions, referrals, discharge notes, electronic data exchange, reimbursement, etc. The current study used a part of the narrative module and the ICPC classification register only.

**Figure 1:**
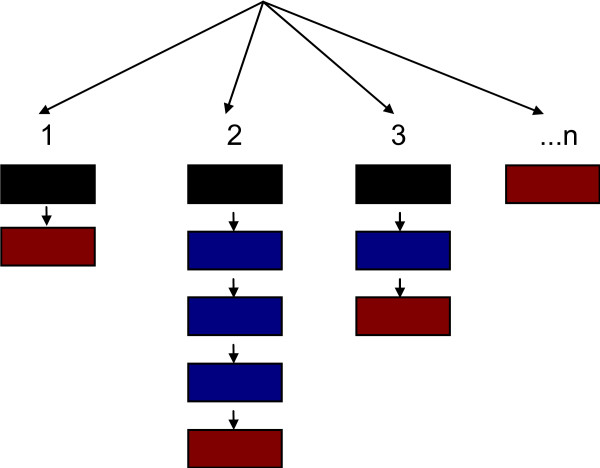
**The tree structure of PROMED with '1 to n' problem histories**. Each box represents one database record for a problem event. Problem 1 has two events; problem 2 has 5 events, etc. The history of each problem starts with a black box and ends with a red; the blue are intermediate events.

**Figure 2:**
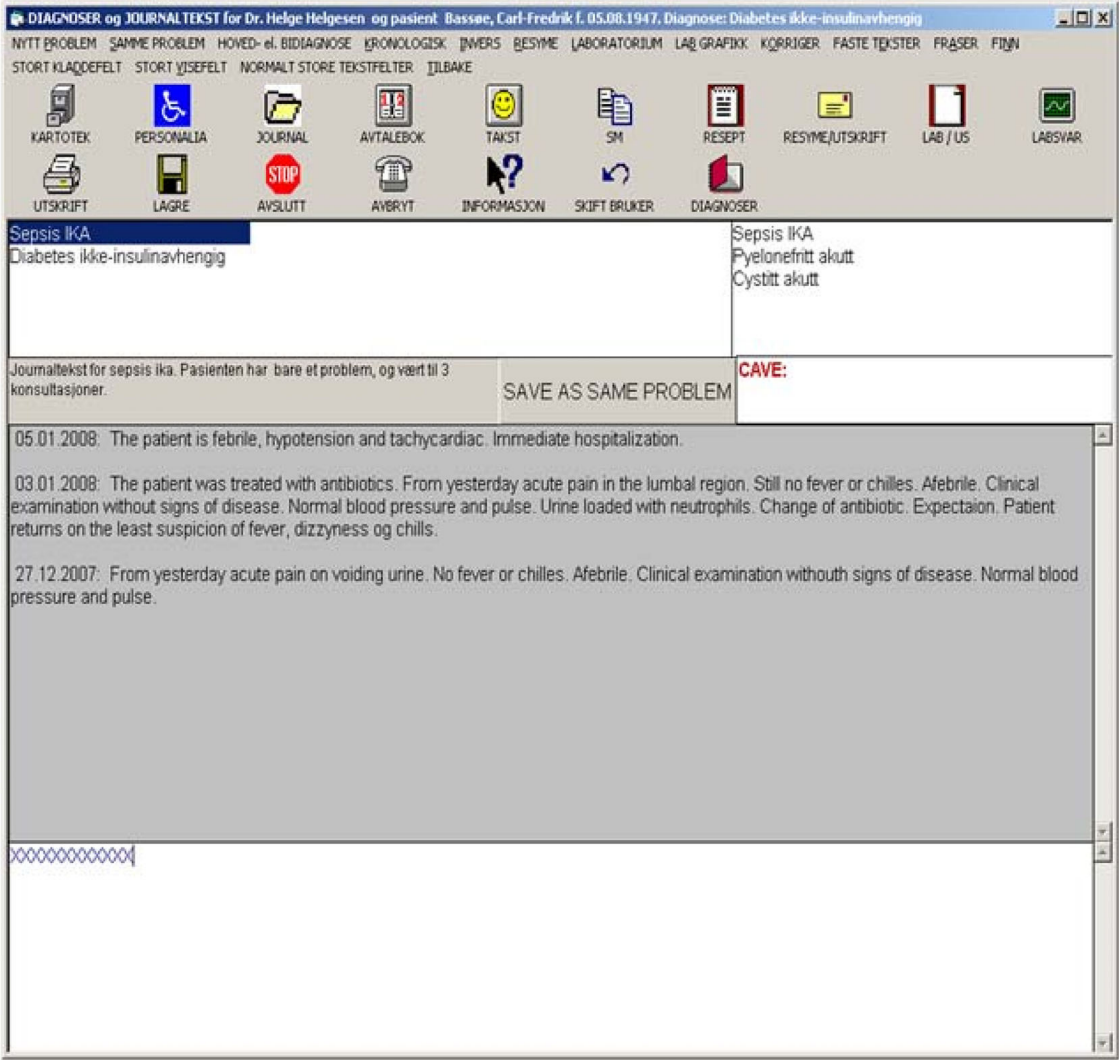
**Diagnoses and narratives for a patient with two problems**. The problem 'septicaemia' (i.e. 'Sepsis IKA') is highlighted in blue (upper left list). The history of the problem events before 'septicaemia' is shown in the upper right list. All narratives for 'septicaemia' problem are shown in the text field with the grey background. The lowermost field with the white background contains the narrative ('XXXXXXXXXXXX') to be added. An ICPC diagnosis/code is reused if the physician selects a diagnosis from the upper left list and presses the button 'SAVE AS SAME PROBLEM'. Otherwise a new diagnosis-code is selected from the ICPC diagnosis-code register through the 'NYTT PROBLEM' (translation: 'NEW PROBLEM') menu. The icons above the two lists give access to other modules and automatically change the menu options

The problem-oriented conceptual design of PROMED is shown in Figure 1. Each record in the narrative module defines one problem event (single coloured boxes; Figure 1). Problem events are implemented as database records; each record stores a narrative with the diagnosis and the corresponding ICPC code and is also stamped with the author's identity, the time, and other parameters. A problem history is defined as a sequential list of problem events and the last diagnosis in a problem history (red boxes; Figure 1) is the problem name; problem names are displayed separately from the problem histories (Figure 2). For example all the events for a patient's diabetes problem could be stored in one problem history.

It should be noted that physicians were free to store clinical narratives for pneumonia, pregnancy and hypertension in separate problem histories. They also decided how to partition their patients' problems and how to organize the list of events during the consultation. Subsequently, the physicians' overall feedback determined the evolution of the PROMED structure.

The first time an ICPC diagnosis/code is used for a new problem (black boxes; Figure 1) the corresponding diagnosis and code is selected from the ICPC register (see below). Considering that problem histories may evolve, e.g. acute cystitis→acute pyelonephritis→septicaemia, whenever a problem's diagnosis or code is changed, the new ICPC diagnosis and code is selected from the ICPC register again.

Diagnoses and codes associated with events can be reused, typically when a new narrative on diabetes, i.e. a new diabetes event, is added to the diabetes problem; an example is shown in Figure 2. Here, the patient has two problems: septicaemia, which is selected (highlighted in blue in the left list; Figure 2) and diabetes. All the stored narratives for septicaemia are displayed in the middle grey field. The problem history for the selected problem is displayed in inverse chronological order (three problem events in the right list; Figure 2). When an event is selected in the problem history list only the narratives corresponding to this event are displayed (middle grey field; Figure 2). In order to reuse a diagnosis-code combination the physician has to select an event from the problem history list and press the button 'SAVE AS SAME PROBLEM' (Figure 2).

**Figure 3:**
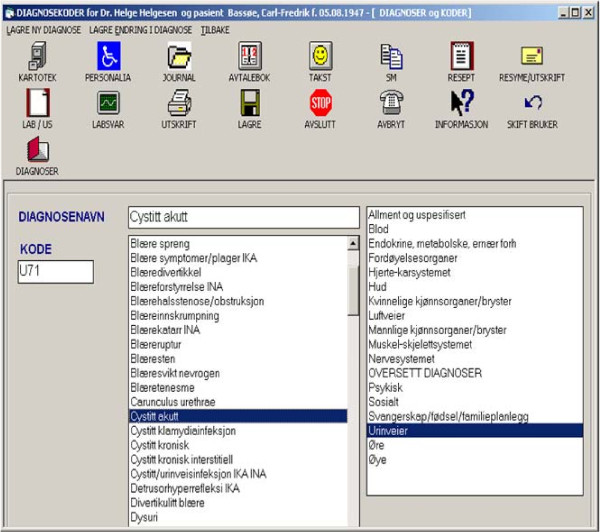
**User interface of the ICPC diagnosis-code module**. Users may select a category from the right list and then the corresponding ICPC-2 diagnosis from the left list. Alternatively, the diagnoses may be selected using substring search in the 'DIAGNOSENAVN' (translation: 'DIAGNOSIS NAME') field. In both cases, the selected ICPC-2 code and diagnosis are automatically assigned to global memory variables and are used in all the PROMED modules. For example, the diagnosis acute cystitis (i.e. 'Cystitt akutt') is selected (highlighted blue) and is automatically copied to the 'DIAGNOSENAVN' field; the corresponding ICPC-2 code is automatically copied to the 'KODE'(translation: 'CODE') field.

ICPC diagnoses and codes for new problem events are selected as it is shown in Figure 3. After their selection from the ICPC register they are automatically assigned to clinical narratives (as described above) and are also used in a variety of other contexts and modules of PROMED. A 'new' diagnosis that does not exist in the ICPC register can be associated with an existing code by adding a new ICPC diagnosis/code record in the register. This is accomplished by selecting a code (or diagnosis), renaming the diagnosis in the field to the right of 'DIAGNOSENAVN' (Figure 3) and adding the record by using the menu choice 'LAGRE NY DIAGNOSE' (translation: 'SAVE NEW DIAGNOSIS').

In 2004 all problem events that contained ICPC-1 all codes were automatically updated to meet the latest Norwegian ICPC-2 coding schema using a data conversion file that was provided by KITH. Nevertheless, it should be mentioned that the diagnosis texts were not converted.

As aforementioned, KITH extended the English ICPC-2 version and provided physicians with more than one diagnosis text options per code. Particularly, the ICPC-2 Norwegian version contains 6390 alphanumeric codes with synonyms, specifications and extensions of the original diagnoses that are included in the English ICPC-2 version, as well as other special terms that physicians used frequently to cover their clinical needs. For example, 'D01' code corresponds to the 'Abdominal pain/cramps general' diagnosis text field in the English version; the same code corresponds to 11 diagnosis text field options in the Norwegian version (Table 1). There are codes with even more options than that, for example:

**Table 1:** Code 'D01' corresponds to the 'Abdominal pain/cramps general' single diagnosis in the English ICPC-2 version and the ICPC-1 Norwegian version; the same code corresponds to 11 text field options in the ICPC-2 Norwegian version, as it is shown in the first column of the table.

Diagnosis text field (Norwegian ICPC-2)	Diagnosis text field (English translation)
Abdomen symptomer/plager INA*	Abdomen symptoms/complaints INA

Abdominal ømhet	Abdominal tenderness

Abdominalsmerte INA*	Abdominal pain

Abdominalsmerte/krampe generell	Abdominal pain/cramp general

Akutt abdomen	Acute abdomen

Kolikksmerter	Colic pain

Magesmerter akutt	Stomach acute pain

Magesmerter uspesifikke	Stomach pain, unspecified

Smerte abdomen uspesifikk	Pain abdomen unspecified

Spedbarnskolikk	Infant colic

Tremånederskolikk	Three month colic

The English translation was added by the authors to allow a better comprehension of the extra options that are offered to the Norwegian users; these options are not available in the English ICPC-2 version.

*INA: not further specified (Ikke Nærmere Angitt).

• L99 ('Musculoskeletal disease other') with 167 options,

• T99 ('Endocrine/metabolic/nutritional disease other') with 93 options and

• L82 ('Congenital anomaly musculoskeletal') with 82 options.

Only 104 codes are comparable to codes in the English version and are associated with one diagnosis text option only, e.g. X19 ('Postmenopausal bleeding') and W21 ('Concern about body image related to pregnancy'). The brief English ICPC-2 is too limited for creating accurate referrals for specialists, pathologists and radiologists.

The PROMED user group expressed serious concerns about the lack of important diagnoses in the Norwegian ICPC-2 and characterized the existing ICPC-2 diagnosis register as incomplete for clinical and administrative work. Therefore they demanded more diagnoses options than those existing in the ICPC-2 list. Consequently, new routines were embedded in PROMED to allow physicians adding their own diagnosis text still for valid ICPC-2 codes (Figure 3).

### Data Analysis

The data set that was used for the analysis included only the date, the diagnosis and the code fields of the problem events; any patient and physician identifiers as well as geographic origin data were excluded. The final set was delivered by the vendor in accordance with a written agreement from the physicians. The Regional Ethics Committee did not consider the extracted data to contain sensitive information (Ethical approval number: P REK Nord 41/2009).

In this study only problem events from 1992, i.e. the year since ICPC use has been compulsory for all GPs in Norway, were studied. In some PROMEDs the first ICPC event was recorded after 2000. Table 2 shows the first and last consultation dates along with the number of patients and records per site. Six physicians had been using PROMED in Centre 3, and two in each of the remaining centres. Thus, the material contains codes and diagnoses from a total of 19 physicians, 13 males and 6 females; the study covers 254 man-years and a follow up time of 16 years.

**Table 2:** Absolute number and collection period for the extracted consultation data per site (after January 2, 1992)

	First Event Date	Last Event Date	Patients (#)	Problem Events (#)
**Centre 1**	6/1/1992	17/12/2007	9973	304342

**Centre 2**	2/1/1992	15/1/2008	11469	334232

**Centre 3**	2/1/1992	21/12/2007	12179	208139

**GP 1**	2/1/1992	4/5/2007	3758	109129

**GP 2**	1/1/1992	31/1/2008	1337	17925

**GP 3**	8/10/2002	1/5/2007	3809	10512

**GP 4**	2/1/1992	11/9/2007	7800	108394

**GP 5**	2/1/1992	3/9/2007	5010	137635

**GP 6**	8/12/2004	17/1/2006	1140	7971

**GP 7**	3/1/1992	28/12/1999	6025	30500

**GP 8**	6/1/1992	3/12/1999	7077	71004

**GP 9**	9/10/1992	28/1/2008	6374	160811

**Totals**			**75951**	**1500594**

GP: General Practitioner

The official Norwegian ICPC-2 version distributed by KITH served as the basis for analyzing the problem events in each Ms Access database. Specific SQL queries were developed and applied in a 3-step process. In each step all the problem events (records) that passed the previous step were filtered out according to the following criteria:

Step 1: The problem events with an entry in the code field were selected and passed to the next step; records without a code did not enter the next step.

Step 2: It was examined whether the codes of these problem events corresponded to a code in the original ICPC-2 file; only records with valid ICPC-2 codes entered step 3.

Step 3: The problem events from step 2 were queried for their match to the expected ICPC-2 diagnosis text.

The total number of events in all sites (centres and GPs) was the input for the first step; the output was the remaining problem events after applying the appropriate SQL queries. Also, the number of events per site was calculated in each step.

In order to get better insight into the physicians initiative to add new diagnoses, four common clinical problem areas (pneumonia and lower respiratory tract infection, diabetes, tonsillitis and anaemia) were further studied. The events in a problem area, e.g. hereditary haemolytic anaemia and iron deficiency anaemia, were identified using specific SQL queries that contained the appropriate terms and wildcards. Subsequently, the appropriate and inappropriate ICPC use for the four areas was evaluated both for the transition and the regular phase; subsequently, the corresponding frequencies were calculated. SQL queries were also used to study the 'new' diagnoses that were added by the physicians. First, the 'new' diagnoses in the four problem areas were extracted automatically and, second, they were manually evaluated either for the use of synonyms and more specific terms or for the introduction of completely new diagnoses.

SPSS for Windows (version 15.0, SPSS, Chicago, IL) was used for the statistical analysis.

## Results

ICPC codes were missing in 6.2% of all cases (Table 3). Particularly, there was one GP (GP 4) with 36.1% of the problem events having a blank code entry in the corresponding field. Obviously, this was an outlier compared to the percentage of the other sites that ranged from 0% to 7.2%. Problem events with missing codes were observed during the transition phase only since the introduction of a code in PROMED system had been mandatory after 2000.

**Table 3:** Total number of problem events per site and their specific distribution (percentages are calculated over the total number of problem events per site) according to the criteria set in each step.

	Problem events (#)	Missing codes (%)	Code mismatch (%)	Diagnosis text mismatch (%)
**Centre 1**	**304342**	7.2	12.7	49.5

**Centre 2**	**334232**	4.6	2.3	70.0

**Centre 3**	**208139**	0	0.3	69.2

**GP 1**	**109129**	3.8	0.6	6.7

**GP 2**	**17925**	5.3	6.5	24.8

**GP 3**	**10512**	0	5.0	26.4

**GP 4**	**108394**	36.1	0.7	25.1

**GP 5**	**137635**	2.0	0.6	15.4

**GP 6**	**7971**	0	2.3	44.2

**GP 7**	**30500**	6.7	2.6	77.7

**GP 8**	**71004**	3.9	5.6	73.6

**GP 9**	**160811**	2.6	2.8	82.1

**Totals**	**1500594**	6.2	4.0	53.8

GP: General Practitioner

Code entries did not always correspond to a correct ICPC-2 code. A mismatch appeared in 4.0% of the total problem events (Table 3). Excluding Centre 1, which is an outlier with 12.7% mismatch, the range for the remaining was between 0.3% and 6.5%. Also, the percentage of problem events with correct ICPC-2 codes was 89.8%. This high value can be explained by the fact that physicians had to use valid codes in order to be reimbursed for their services after 2000.

In most cases, event diagnoses did not match the standard ICPC-2 text (53.8%; Table 3). Particularly, in three sites (GP 7, GP 8 and GP 9) the percentage was remarkably high (up to 82.1%) while lower (but still high) in the rest. Summarizing the results, only 36.0% of the approximately 1.5 million problem events met all the criteria and included a valid ICPC-2 code followed by the correct ICPC-2 diagnosis.

**Figure 4:**
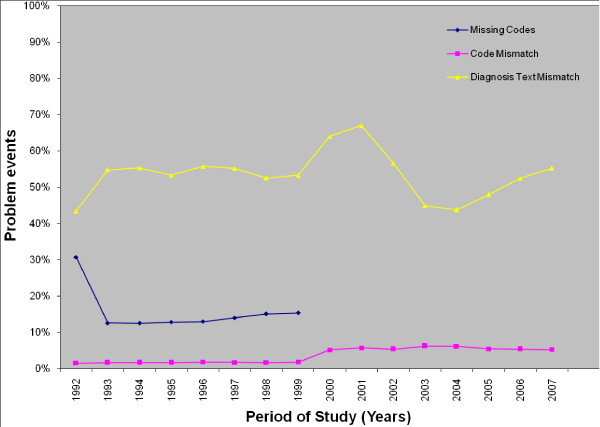
**The percentage of problem events with missing codes, code mismatch and diagnosis text mismatch over the total number of problem events per year**.

The percentages for the three categories (problem events with missing codes, code and diagnosis text mismatch) over the total number of problem events per year are shown in Figure 4. Generally, the percentage of missing codes was stable from 1993 to 1999; the code mismatch rate was low and stable during the transition phase while slightly higher but still stable during the regular phase. The diagnosis text mismatch rate dropped from 2003 to 2004 but increased thereafter. This indicates that physician's attitude towards ICPC standard did not change significantly over the 16-year period of study not even after the introduction of the ICPC-2 version.

The identification of invalid ICPC-2 diagnoses shows that physicians added 'new' diagnoses to their local ICPC-2 database and used them to classify the problem events. 'New' diagnoses may have been used once, a few times or repeatedly. For example at Centre 1, 3834 'new' diagnoses were used for 49.5% of the problem events; interestingly, 793 of them were used only once, while remarkably less (<50) were reused for more than 10 times.

Moreover, the introduction of 'new' diagnoses was examined specifically for four clinical problem areas. The number of different 'new' diagnoses for pneumonia, diabetes, tonsillitis and anaemia were 56, 114, 78 and 89 respectively. In the case of pneumonia names of microorganisms (mycoplasma, hemophilus influenza, pneumococcal, bacterial), time sequence (acute, relapsing), process (control, observation), anatomical site (right side) and consequence (sequel of) were added.

The KITH ICPC-2 version contained 15 entries with the substring 'tonsi'. Relapsing events and information on treatment were found in the tonsillitis events, however not in the ICPC-2 standard. Also, the ICPC-2 register has an entry for streptococci, which was spelled differently in the examined events. Additionally, tonsillitis was combined with mononucleosis in the 'new' diagnoses, but not in KITH's ICPC-2 version; surprisingly, there were no specifications of mononucleosis in the standard ICPC-2.

Regarding diabetes the KITH ICPC-2 version had 25 main entries and four more for glucose-related problems. Glucosuria was diagnosed in the examined problem events, but the corresponding entries in ICPC-2 included the descriptive term 'sugar in the urine'. In some cases two main problem events were combined in the diagnosis field, e.g. anaemia and diabetes.

The standard ICPC-2 has 25 entries containing the substring 'anemi' most of which were also spelled differently in the examined events. Also, ICPC-2 does not contain information for either the degree, e.g. 'severe', or the cause of bleeding, e.g. 'hypermenorrhoea'; this information was found in the 'new' diagnoses for anaemia.

In all the problem areas, the uncertainty of diagnosis was stated by a question mark; the differential diagnosis was denoted by the inclusion of clinical problems having similar symptoms and signs. The physicians had used many more specific terms and aspects that did not exist in ICPC.

The use of appropriate and inappropriate ICPC-2 codes for pneumonia and lower respiratory tract infection, anaemia, tonsillitis and diabetes is shown in Table 4. Generally, inappropriate codes were used in both periods for the four problem areas; only in a few cases these codes were 'corrected' after 2000 while new inappropriate 'incidents' appeared. It should be noted that the inappropriateness was not due to mismatches in the ICPC-1 to ICPC-2 conversion table.

**Table 4:** The problem events with appropriate and inappropriate ICPC codes (before and after 2000) for the four problem areas.

		Pneumonia		Diabetes		Tonsillitis		Anaemia	
		
		A	I	A	I	A	I	A	I
	**Problem events (#)**	3419	5	9450	935	4081	34	5248	35
	
	**% of the total events per area**	95.8%	0.1%	84.8%	8.4%	96.5%	0.8%	94.7%	0.6%
	
**Before 2000**	**Median ± SD**	444 ± 93	0 ± 1	1222 ± 449	114 ± 16	543 ± 107	13 ± 4	702 ± 181	0 ± 11
	
	**Mean ± SE**	427 ± 32.9	1 ± 0.5	1181 ± 158.7	117 ± 5.6	510 ± 37.8	4 ± 1.4	656 ± 64.1	4 ± 4.0
	
	**Range**	228-544	0-4	390-1628	96-148	324-645	1-13	250-851	0-32
	
	**Problem events (#)**	5411	11	17874	2222	3982	98	8898	6
	
	**% of the total events per area**	99.6%	0.2%	88.9%	11.0%	97.3%	2.4%	99.7%	0.1%
	
**After 2000**	**Median ± SD**	611 ± 199	1 ± 2	2150 ± 381	263 ± 58	442 ± 128	13 ± 4	1138 ± 109	1 ± 1
	
	**Mean ± SE**	676 ± 70.5	1 ± 0.6	2234 ± 134.8	278 ± 20.4	498 ± 45.2	12 ± 1.5	1112 ± 38.4	1 ± 0.4
	
	**Range**	447-1011	0-4	1837-2802	216-402	366-667	6-19	883-1247	0-3

Mean, median and range were calculated per group of problem events.

A: Problem events with appropriate ICPC codes for pneumonia (R78, R80, R81, R95, R99); diabetes (A91, F83, F92, L99, N94, T87, T90, T99, U99, W84); tonsillitis (R72, R76, R90); and anaemia (A85, B74, B78, B79, B80, B81, B82, B99, T91).

I: Problem events with inappropriate ICPC codes for pneumonia (D01, U88, R74, R96); diabetes (D01, D11, D70, K77, F29); tonsillitis (D01, R21, R24); and anaemia (D01, L84).

SD: Standard Deviation; SE: Standard Error of the Mean.

## Discussion

The present study shows that a low percentage (only 36.0%) of the codes and diagnoses that were assigned to problem events agreed with the ICPC-2 standard; mismatches were observed at all primary care sites. Our results agree with Tai *et al *who reported that current systems for clinical coding promote the diversity rather than the consistency of clinical coding [9]. Clinical practice requires accurate diagnoses that reflect the patients' clinical problems. Standards like ICPC are thought to facilitate clinical research, administrative work, epidemiological studies and information exchange between computerized health care systems within the same or different countries. However, the reduction of diagnostic options to the 684 crude classes of the English ICPC version ignores not only the complexity of clinical problems, but also the necessity for accurate information. Our results show that physicians demanded and actually used significantly more diagnoses than the 6390 of the Norwegian ICPC-2. Thus, it is strongly suggested that even the extended ICPC-2 is inappropriate for clinical work.

The physicians created many 'new' diagnoses and assigned them to problem events. In the four selected problem areas, the 'new' diagnoses covered various aspects such as time, progression, degree, aetiology, anatomical sites, treatments and complications; they also stated the uncertainty in diagnosis and included the differential diagnosis if needed. Even though this is a small subset of only four clinical problems, it is obvious that ICPC is missing important diagnostic information. These findings also reveal the fundamental problem with the structure of ICPC (also met in ICD): if diagnoses were presented as one list on the basis of systematic combinations of dimensions (e.g.100 body regions, 5 labels for time course dimension, 10 aetiology agents, 10 pathogenetic mechanisms, 5 degrees of severity, etc.) there would be a long list including millions of elements. The appropriate way (as in SNOMED) could be the selection of one element from each dimension and the construction of a diagnosis [10-12].

The PROMED functionality that allowed the modification of codes and diagnoses might appear to introduce a limitation in our study. However, this should not be attributed to the PROMED system but rather to the fact that physicians actively created and assigned the appropriate diagnoses to the problem events when they were not available in ICPC. Considering that this required additional work load it could be hypothesized that physicians would avoid giving incorrect diagnostic labels to their patients if they had an alternative. It is also obvious that their primary concern was to avoid patients' misclassification, which could lead to wrong treatments and/or inappropriate diagnoses on referrals or sick certificates.

The low number of records with code mismatch compared to the number of records with diagnosis text mismatch was expected given that correct ICPC codes were required for reimbursement purposes. Problem events without a code occurred during the transition phase only, when the PROMED system incorporated ICPC-1 version and allowed the recording of an event without a code. Even though this was expected, it consists an important finding since it underlines the necessity for EPRs to disallow the lack of codes.

It could be argued that our results are not representative of the ICPC use in primary care. However, the problem events that were investigated (approximately 1.5 million) reflect the demands for diagnoses and codes over a huge number of problem events and for a long period of study; the number of physicians involved is also sufficient. Even though these numbers strongly suggest that our results are representative for ICPC use, further studies are required to validate our findings.

Letrilliart *et al *concluded that when software incorporates large terminologies, physicians will use it only if they are special trained and rewarded [13]. In this context, it might be argued that our physicians were not appropriately trained. It should be mentioned though that they were all trained adequately and were provided with paper-based and online manuals; additionally, the correct use of ICPC was rewarded. Thus, the lack of training and reward is not a solid argument for the validity of our results.

Jordan *et al *reported that GPs have personal preferences for certain codes, which are not always appropriate, and that they feel pressured to use them even if the codes are not correct for a patient case [14]. The physicians in our cohort decided to put extra effort in order to accomplish the task of adding 'new' diagnoses even though they used most of them only once. We foresee two alternative solutions for this problem. The first is to allow physicians adding diagnosis-code combinations when necessary, as in PROMED; unfortunately, such an approach would ruin the standard. The other alternative is the development of a well-structured dimensional classification like SNOMED, but such a classification should have a solid structure based on clinical practice.

All major classifications, e.g. ICD, ICPC and SNOMED are currently undergoing (major) international revisions. This indicates either problems of structure or problems of content as it was shown in the current study. Full insight into the reasons for the inappropriate use of codes and diagnoses would require a thorough analysis of their documentation in laboratory results, clinical narratives and elsewhere. This could be accomplished only in a dedicated research project that would incorporate full access to patients' data as well as Natural Language Processing (NLP) and other advanced computerized techniques; this is definitely beyond the goals of the current study.

## Conclusions

Standards like ICPC are supposed to facilitate clinical research, development, epidemiological studies and data exchange. However, our results strongly suggest that ICPC is inappropriate for clinical work and raise serious objections against its applicability. An in depth revision of ICPC-2 or possibly an entirely new approach is needed. We suggest a combinatorial approach (as in SNOMED), but this would require a complete reworking of ICPC structure. Whether the barriers to such a direction can be overcome remains to be investigated.

## Competing interests

The authors declare that they have no competing interests.

## Authors' contributions

TB has participated in the study design and the preparation of the manuscript, he has also performed the data analysis; C-FB developed and maintained PROMED, participated in the study design and the preparation of the manuscript; GH supervised the study. All authors read and approved the final manuscript.

## Pre-publication history

The pre-publication history for this paper can be accessed here:

http://www.biomedcentral.com/1472-6947/10/11/prepub
